# Effectiveness-implementation hybrid trial of Spanish language, digital cognitive-behavioral therapy (dCBT) intervention for depression and anxiety – protocol for the SUPERA (SUpport from PEeRs to expand Access) study

**DOI:** 10.1016/j.cct.2023.107422

**Published:** 2023-12-24

**Authors:** Adrian Aguilera, Marvyn R. Arévalo Avalos, Karina Rosales, Yazleen Reyes, Rosa Hernandez-Ramos, Giovanni Ramos, Esmeralda Garcia, Tuyen Hoang, Lisa Ochoa-Frongia, Lisa R. Fortuna, Stephen M. Schueller

**Affiliations:** a School of Social Welfare, University of California Berkeley, Berkeley, CA, United States of America; b Department of Psychiatry, University of California-San Francisco, San Francisco, CA, United States of America; c Department of Psychological Science, University of California Irvine, Irvine, CA, United States of America; d Biostatistics, Epidemiology & Research Design (BERD) Unit, University of California, Irvine, Irvine, CA, United States of America; e School of Medicine, University of California-San Francisco, San Francisco, CA, United States of America

**Keywords:** Digital mental health, Cognitive-behavioral therapy, Depression, Anxiety, Peer-support, Latinx

## Abstract

**Background:**

Limited English Proficiency (LEP) Latinxs experience a longer duration of untreated depression and anxiety. LEP Latinxs have difficulty accessing mental healthcare due to insufficient Spanish-speaking behavioral/mental health clinicians to meet demand. These under-resourced healthcare systems are less likely to be the site for the implementation of innovations. Digital interventions can provide an effective option for overcoming these barriers; yet, when digital evidence-based treatments are available, uptake and engagement is often low. This manuscript presents the protocol for the *SUPERA* (SUpport from PEeRs to expand Access) study which will evaluate the implementation of an evidence-based, Spanish language, digital cognitive-behavioral therapy (dCBT) intervention (i.e., SilverCloud) in safety-net primary care clinics for LEP Latinx patients with depression or anxiety.

**Methods:**

We will conduct an effectiveness-implementation hybrid trial (Type 2) design comparing engagement and clinical outcomes in two modalities of dCBT delivery (peer-supported vs. unsupported). We will also compare provider-level outreach (using a clinic patient registry) versus inreach (traditional provider referral) to compare rates of initiation, completion, and cost. Participants will be 426 LEP Latinx adults ≥18 years of age, PHQ-9 ≥ 10 or GAD-7 ≥ 8, with access to the internet via smartphone, and not currently receiving individual psychotherapy. We will collect baseline, post-intervention (8 weeks), and follow up (3 months) data.

**Conclusion:**

The long-term goal of this research is to aid in the implementation of digital mental health interventions that can be sustainably implemented in low-resourced settings, while reducing the reliance on professionals, overcoming workforce deficits, and increasing relevance for diverse populations.

## 1. Background

The Latinx population comprises one of the largest (~62.5 million) racial/ethnic groups in the US [1], with approximately 16% of those who report speaking English less than “very well” or “not at all” [2]. Latinxs experience high prevalence of depression [3] and anxiety [4] at 8.2% and 14.5% respectively. Compared with their English-speaking counterparts, Latinxs with limited English proficiency (LEP) with depression or anxiety disorders wait longer to receive treatment and when they do, they typically attend fewer sessions [5] and receive a lower quality of care [6,7], which negatively affects clinical outcomes [8,9]. Reasons for lower engagement among LEP Latinxs include a lack of Spanish-speaking providers [6] and unsatisfactory interactions with providers due in part to cultural and linguistic differences [10]. Although efforts have been made to integrate behavioral health into primary care, a severe paucity of Spanish-speaking trained clinicians makes doing so in a culturally-congruent manner challenging [6]. Multiple innovations, such as the use of digital technology and peer support [11], in mental healthcare could help alleviate these problems. However, safety-net healthcare systems, where many LEP Latinx receive their care [12], are often under-resourced and less likely to serve as sites for implementation of innovations [13]. When they do receive the option, LEP Latinx patients report high satisfaction with culturally adapted digital interventions [14].

Over the past decade, an increasing number of mental health interventions have integrated digital technologies to provide efficacious treatments [15]. As a result, digital treatments are recommended as frontline treatments for mental health issues including depression and anxiety [16,17]. Yet, few patients have access to evidence-based digital treatments as part of their care, and even when available, uptake and engagement –x both by providers and patients – is often low [18]. Research is needed to develop and evaluate implementation strategies to support the integration of digital mental health into healthcare settings [19], especially in locations where access to evidence-based care is limited for several structural reasons.

Digital interventions are especially relevant for low-resource settings, as they are scalable and can deliver evidence-based interventions with fidelity while minimizing the need for specialty mental health providers [20]. Previous evidence has shown that human support, both by professionals and non-professionals, can increase the uptake and effectiveness of digital mental health interventions [21,22]. Non-professional peers belonging to local community organizations may be able to provide linguistically- and culturally-congruent support and be more cost-effective and feasible than providing professional support. Some studies have assessed various task shifting approaches in international contexts with limited professional providers [23]. More work, however, is needed to determine how such models can inform models of support for digital interventions in ways that would make providing support more feasible while effectively increasing benefit and engagement.

**Fig. 1.**
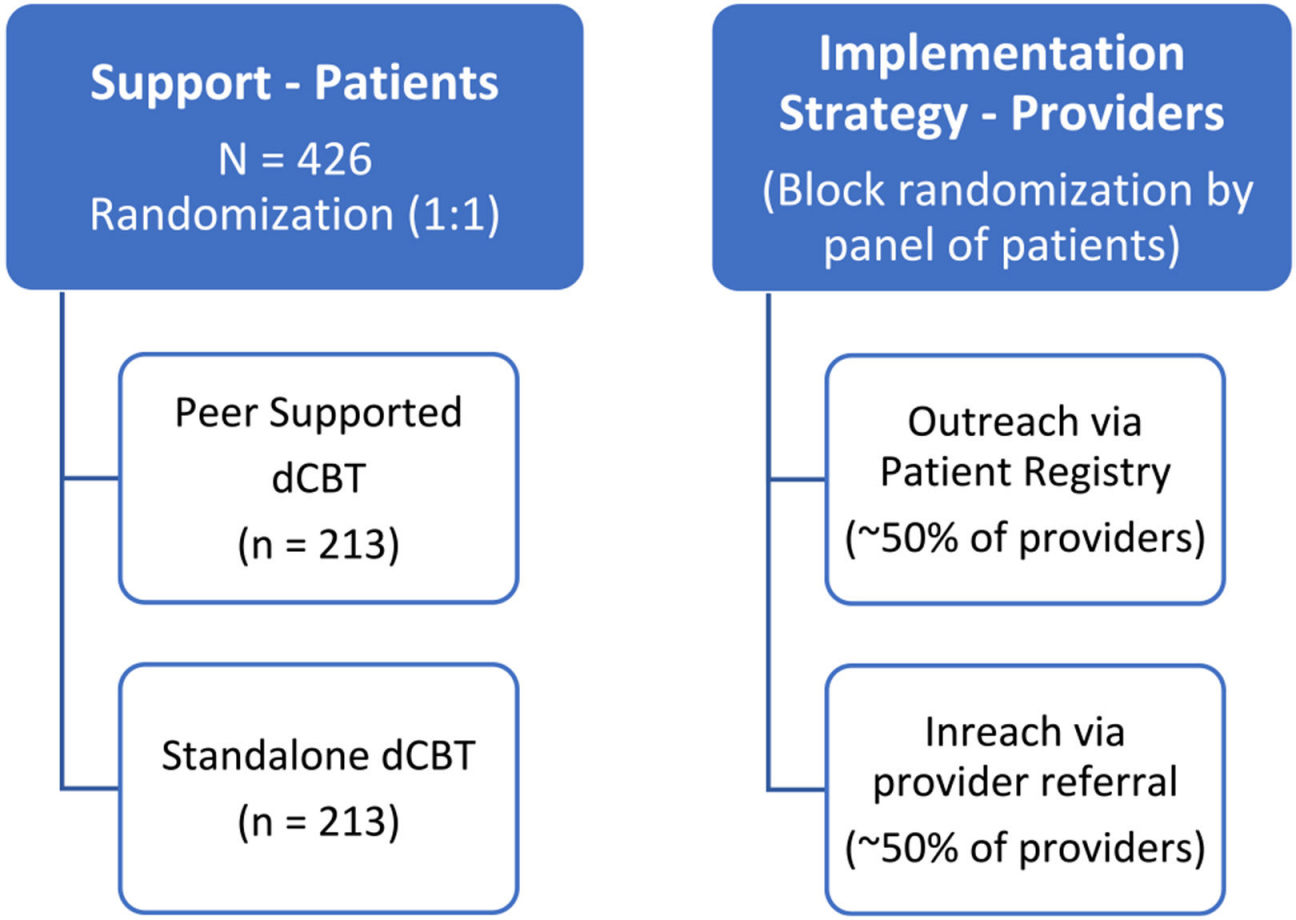
Randomization design.

In the proposed SUPERA (Support from PEeRs to expand Access) study, we will evaluate the implementation of an evidence-based, Spanish language, digital cognitive-behavioral therapy (dCBT) intervention (i.e., SilverCloud) [24] in safety-net primary care clinics for LEP Latinx patients with depression or anxiety. We will conduct an effectiveness-implementation hybrid trial (Type 2) [25] design with both provider- and patient-level randomization (Fig. 1). At the patient level, we will compare two modes of delivery of the dCBT platform: peer-supported and unsupported. At the provider level, we will compare outreach (direct-to-consumer using clinic patient registry) with inreach (traditional provider referral).

## 2. Aims and hypothesis

The SUPERA Study specific aims include:

### Aim 1:

Evaluate effectiveness of dCBT modalities. Enrolled participants will be randomized to a two-armed trial comparing peer-supported vs. unsupported dCBT (SilverCloud). *We hypothesize that peer-supported dCBT will result in greater improvement in mental health symptoms and higher patient engagement compared to unsupported dCBT*.

### Aim 2:

Evaluate impact of outreach vs. inreach on implementation outcomes. We will use block randomization to assign blocks of providers to a two-armed trial comparing outreach vs. inreach. Implementation evaluation will follow the RE-AIM model (See Table 1.) The primary outcome will be reach as defined by the proportion of eligible patients referred and initiating dCBT comparing outreach vs inreach strategies. *We predict an outreach (registry based direct-to-consumer) strategy will result in more patients referred, initiating and lower relative cost compared to inreach (provider referral,) which is likely to result in a higher percentage of patients participating.*

### Aim 3:

Evaluate putative mechanisms of change for the intervention and implementation strategies. We will conduct a mixed-methods evaluation consisting of surveys, interviews, and focus groups. *Aim 3a):* We will include patients and peers to assess attitudes towards the intervention, support component, cultural relevance as well as relationship factors and CBT skills use, knowledge, and fit. *Aim 3b):* We will include clinic leadership and providers to assess climate, clinic readiness, and attitudes towards the intervention, including potential for sustainability. This will provide rich contextual and process information for the implementation evaluation.

## 3. Materials and methods

### 3.1. Participants and procedures

This is a type 2 effectiveness-implementation hybrid study with both provider- and patient-level randomization. Thus, it includes simultaneous evaluation of effectiveness and implementation aims, allowing to test both the treatment effects of dCBT on patient outcomes and the impact of implementation strategies (inreach vs. outreach) on implementation outcomes.

#### 3.1.1. Aim 1 participants, recruitment, and enrollment procedures

Eligibility criteria include 1) clinically significant symptoms of depression or anxiety (i.e., PHQ-9 (Patient Health Questionnaire-9) ≥ 10 or GAD-7 (Generalized Anxiety Disorder 7) ≥ 8, similar to other studies of dCBT [26] and recommendations of cutoffs on these questionnaires^27,28^; 2) access to the Internet via smartphone or broadband at home; 3) a basic level of digital literacy (as measured by the UCSF CVP Questions to Screen Patient Digital Needs 6 item self-reported questionnaire) [29] or willingness to undergo a brief adapted digital training appropriate to patient needs; 4) ≥18 years of age; 5) preference for receiving medical care in Spanish. Exclusion criteria include 1) currently receiving formal psychotherapy, however, participants are allowed to be referred while participating in this study; 2) visual, hearing, voice, or motor impairment or low literacy that would prevent completion of study procedures; 3) diagnosis of a psychotic disorder, bipolar disorder, dissociative disorder, or substance use disorder; and 4) severe active suicidality (i.e., endorsing suicidal ideation with plan and intent). Patients will be required to provide informed consent prior to study enrollment. Eligibility criteria will be determined through self-report and verified through participants’ electronic medical records to ensure accuracy.

We will recruit and enroll 426 patients receiving care from safety-net primary care clinics within the San Francisco Health Network (SFHN). Once a patient has preliminarily agreed to participate, they will be asked to complete an in-person or phone-based eligibility screen conducted by the research team. All eligible participants will be randomized (1:1) to delivery conditions described below. Participants will remain eligible for eight weeks before having to undergo a new eligibility screen before beginning treatment. Participants will complete baseline study measures immediately prior to beginning the intervention. Enrolled participants will receive a brief technology use training covering basic skills in digital literacy, how to login and use the SilverCloud intervention platform, the basic features of SilverCloud, and how to contact the study team for technical support.

#### 3.1.2. Aim 2 participants and procedures

We will announce the study during regular primary care and behavioral health provider meetings and through direct emails to providers. Providers will be randomized such that their panel of patients will either be in the outreach or inreach condition.

#### 3.1.3. Aim 3 participants and procedures

We will use data collected under Aims 1 and 2 to identify patient participants who were referred but did not initiate, those who initiated but did not benefit, and those who initiated and did benefit, balanced across treatment arms (Aim 3a). For Aim 3b, we will identify key informants including clinic leadership and providers.

Patients will be recruited by direct messaging from our research team. Participants who did not initiate will receive an invitation to participate one-month post-referral. For participants who did initiate treatment, benefit will be defined as those who have improved at end-of-treatment (week 8) using a standard definition of definition of PHQ-9 < 10 or GAD-7 < 8 and at least 50% improvement from pre-treatment score [30]. Participants meeting these thresholds will be contacted immediately after completing the program (week 8) and interviews will be scheduled to occur within one month of treatment completion. Recruitment will continue until saturation is achieved using a thematic analysis [31]. We anticipate conducting approximately 10–15 interviews for those who did not initiate treatment, 10–30 for those who did not benefit, and 10–30 for those who did benefit, which will allow us to balance across the two study arms of supported and unsupported dCBT [32].

Providers and leadership will be recruited through direct emails, listservs, and presentations at staff meetings. Participants need to hold a leadership or provider role in one of the primary care clinics where recruitment occurred but need not be providing care to a study participant. We anticipate provider focus groups will range in size depending on provider availability. We will also conduct interviews when necessary to obtain key informants, especially clinic leadership. Recruitment of providers/leaders will continue until saturation is achieved using a thematic analysis. We anticipate this will result in 10–30 providers/leaders recruited depending on variations in performance and themes across clinics.

We will conduct individual remote interviews following a semi-structured interview guide created using the CFIR (Consolidated Framework for Implementation Science) interview guide tool [33,34]. Individual interviews were selected rather than focus groups to minimize any anxiety or embarrassment that may arise based on a person’s experience or views and also because we are selecting from three potential groups of patient participants based on their experience with the program (did not initiate, did not benefit, benefited). These interviews will last approximately 45 min to an hour and participants will be compensated for their time. All interviews will be audiotaped.

**Fig. 2.**
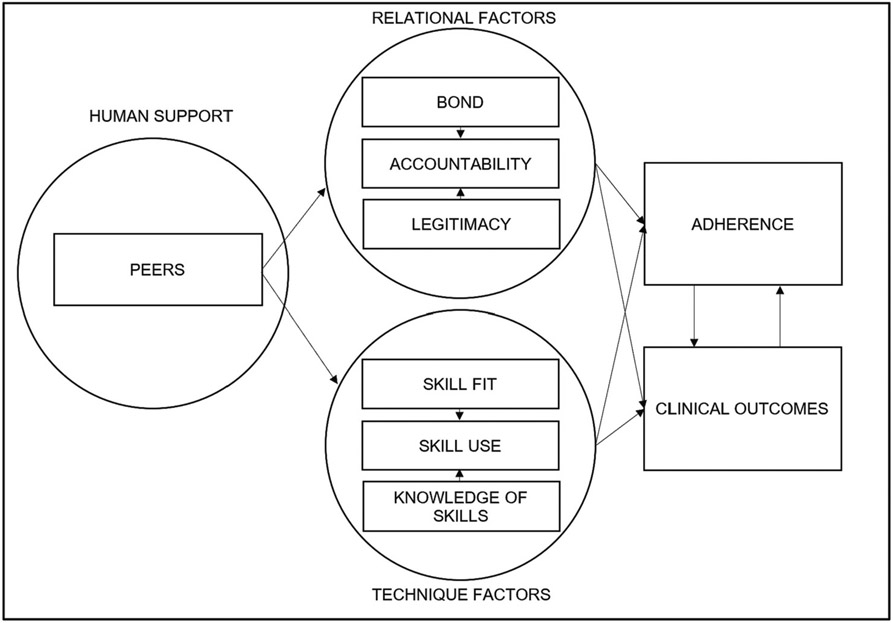
Conceptual model combining supportive accountability and efficiency model.

Participants in the trial will receive additional questionnaires (See Table 2) to determine putative mechanisms according to an experimental therapeutics approach [35]. In line with our conceptual model (see Fig. 2), these mechanisms include relational factors including bond, acceptability, and legitimacy and technique factors including CBT skill use and knowledge of CBT skills.

Interviews will last approximately 45 min to an hour and participants will be compensated for their time. These interviews will identify characteristics of the intervention, individuals involved, setting, and implementation processes. Additionally, this interview guide will address aspects related to identifying mechanisms important for sustainability of peer-supported dCBT. All attendees of the focus groups and interviews as well as all providers responsible for any patient who participates in this trial will receive a brief survey link via REDCap including the provider/leadership survey measures (see Table 2).

### 3.2. Study conditions

#### 3.2.1. Aim 1 study conditions

All participants will receive free access to the dCBT intervention, which is a Spanish language and culturally-adapted version of the SilverCloud *Space from Depression & Anxiety* (comorbid) program. SilverCloud was selected because it has been used by over 500,000 users, has been involved in multiple trials, including efficacy trials, pragmatic trials, and trials that used culturally- and linguistically-adapted versions [36-39]. SilverCloud has been adopted and deployed in major health systems including the UK’s IAPT program and Kaiser Permanente in the US [40-42]. Within the program, users progress through a series of psychoeducational modules covering the CBT principles for the treatment of depression and anxiety and intended as an 8-week treatment. SilverCloud is available on both web and mobile devices. Participants will be instructed to use this program for eight weeks.

##### 3.2.1.1. Community peer-supported dCBT.

In the peer-supported dCBT condition, each participant will be assigned a Spanish-speaking peer supporter who will provide regular support based on our coaching support protocol. Supporters will conduct a brief engagement call (30–40 min) to introduce themselves, orient the participant to the role of the peer supporter, provide an overview of SilverCloud, and identify goals and set expectations for this program. Supporters will then provide weekly check-ins via messaging or phone calls. The goals of weekly check-ins are to identify and resolve potential barriers outlined in the Efficiency Model [43] (usability, engagement, knowledge, fit, and implementation) and to monitor symptom severity and progress. Participants will be able to communicate with supporters through the dCBT platform.

Community peer-supporters will be recruited from a local community organization. Interested supporters will participate in training led by the research team. The supporters will be trained on our coaching support protocol [27-29] that details instructions for conducting an engagement phone call, monitoring participants’ use of SilverCloud (i.e., the frequency, content, examples of weekly messages) and handling FAQs, crises, and escalation of clinical need. Supporters will receive a coaching manual that includes description of the key features of SilverCloud, procedures for the engagement phone call and follow-up messaging, including examples of how to conduct these sessions and sample messages. SilverCloud also provides message templates for supporters to send to participants. Supporters will support a maximum of five participants at a time and receive regular supervision throughout their involvement in the project.

##### 3.2.1.2. Unsupported dCBT.

In this group, participants will receive the SilverCloud platform without peer support. Participants will receive weekly automated app notifications to encourage engagement.

#### 3.2.2. Aim 2 study conditions

##### 3.2.2.1. Outreach.

Outreach will be a direct-to-consumer implementation strategy that will leverage the patient registry at ZSFG. This registry is based on patient- and provider-reported data and demographics in the EPIC electronic health record (her). Registry reports can be pulled based on validated population health management tools in Epic. Reports include patient demographics, primary care provider problem list diagnoses and visit diagnoses; and structured assessment data including PHQ-9 and/or GAD-7. Outreach will include an initial letter followed by a scripted phone call providing an overview of the study.

##### 3.2.2.2. Inreach.

Inreach will be a clinic-based implementation strategy consisting of a referral by one’s primary care provider or behavioral health team clinician at the time of a usual care visit. In usual care at ZSFG, there are both 1) warm handoffs between primary care and behavioral health clinicians for mental health treatment, and 2) ongoing mental health care provided by the primary care team. In both instances of usual care, we will be able to provide hardcopy materials to enrolled providers to distribute to their panels as the study progresses. These materials will be refreshed by research staff attending regular clinical meetings on a monthly basis.

### 3.3. Measures

#### 3.3.1. Aim 1 Measures

All research study assessments will be administered in Spanish. Online assessments will be sent first via our secure online assessment platform (REDCap [44]). Participants who fail to complete the online assessment will be provided a reminder within a one-week window before being contacted by phone with the option of completing the assessment during that call. Assessments will occur at baseline, post-treatment (week 8), and at a 3-month post-treatment follow-up.

##### 3.3.1.1. Primary outcomes.

***Depression and Anxiety.*** The main treatment outcomes are changes in depression as measured by the Patient Health Questionnaire-9 (PHQ-9) and anxiety as measured by the Generalized Anxiety Disorder-7 (GAD-7) from baseline to 8-weeks (which this study is powered on). The PHQ-9 and GAD-7 were selected as the primary self-report outcomes because they are the most widely used measure of depression and anxiety in primary care settings, including the study setting [45]. The PHQ-9 and GAD-7 have been used widely in Spanish-speaking populations as well [46,47].

##### 3.3.1.2. Secondary outcomes.

***Functioning.*** We will use the World Health Organization Disability Assessment Schedule (WHODAS) [48]. The WHODAS includes 12 questions that assess health and disability over six domains: cognitive, mobility, self-care, getting-along, and life activities. The WHODAS has been translated into Spanish and shown good concurrent and divergent validity [49]. ***Patient Engagement.*** SilverCloud allows for passive collection of usage data. Total time spent on the platform will be the metric of patient engagement however other metrics such as number of completed modules will be available.

#### 3.3.2. Aim 2 Measures

Guided by the RE-AIM framework [50], we will measure reach, adoption, and implementation costs (See Table 1.)

***Reach*** will be defined as (a) the number of patients contacted via phone or brochure/text across the inreach and outreach arms and (b) the number of patients who sign-up for SilverCloud over the number of people determined to be eligible. Potentially eligible patients will be identified using the Patient Registry as those having a PHQ-9 ≥ 10 or GAD-7 ≥ 8, a diagnosis of depression or anxiety on their problem list or visit summary, and indicating a preference for Spanish language use. We will also collect demographic information of those contacted and those who sign-up for SilverCloud to compare the cohort of reached patients to demographic characteristics of potentially eligible patients from the ZSFG registry.

***Adoption*** will be the primary outcome defined as the percent of providers with at least one enrolled patient. We will also compare characteristics of providers with at least one enrolled patient on available data such as degree, specialty, and years of practice.

***Implementation Costs.****Patient costs* will include any time associated with engagement in treatment, including time spent on calls, messages, or on the dCBT site. Patient time in treatment will be captured through passive data collection estimating time on site and time of any phone calls that will be audiotaped. Self-reported salary will be used to estimate patient time cost. *Direct intervention costs.* Peers will track time allocated to all support activities, including scheduling, calls, and text messaging. Any scheduled phone sessions missed without 24-h notice will be counted as having occurred as this is a cost incurred. The dCBT costs will also include technical costs, including website maintenance and technical support. These costs will be calculated based on hourly rates for peers and payment made to SilverCloud. This costs analysis will be used to determine this program’s potential for sustainment and help support future work that can look at broader implementation and sustainment.

#### 3.3.3. Aim 3 Measures

Table 2 displays the Aim 3 measures. These will be collected through interviews and surveys with the participants, providers, and clinic leadership. Fidelity monitoring will be conducted through observational naturalistic data collection including contacts made through phone calls, messaging through SilverCloud, and weekly supervision documentation.

### 3.4. Statistical analysis

#### 3.4.1. Aim 1 statistical considerations

A *n* = 426 sample will provide 80% power to detect a 2-point difference in change on the PHQ-9 or GAD-7 scores between the conditions at an *α* = 0.05. This difference was based on previous meta-analytic findings [51], but also provides excellent power to detect changes defined as minimal clinically important differences of 3.7 and 3.3 points for the PHQ-9 and GAD-7 respectively [52]. Power estimates included estimating intra-clinic correlations of 0.3.

We will compare PHQ-9 and GAD-7 scores between the conditions using mixed-effects models with an intention-to-treat protocol. Mixed-effects models generally require three time points which is why we include a post-treatment assessment at 3-months. Although every effort will be made to avoid missing data (e.g., e-mail/text reminders for assessments, financial incentives for completing assessments), mixed-models are robust to missing data. In the case of missing data, we will also analyze if missing data is related to any observable characteristics. Secondary clinical outcomes for this trial will also be analyzed using mixed-effects models. We will also explore baseline severity of symptoms, each supporter, and whether participants initiated other psychosocial treatments as potential moderators of treatment response.

We will compare engagement, as defined as time on platform, across the two arms of the study using implementation strategy as a potential covariate. *Mediational analysis.* We will also explore whether engagement mediates changes in clinical symptoms. Mediation will be examined using a bootstrapping procedure [53]. We will compute different models for activity from users of the platform as well as by supporters of the platform.

#### 3.4.2. Aim 2 statistical considerations

We will compare the proportion of patients referred, initiating, and completing treatment at each of the clinic sites by fitting mixed-effects models using these proportions as continuous outcome variables. These models will allow to control for intraclinic clustering of providers as well as patient characteristics of each clinic. Patient demographics and costs of treatment between arms will be compared using ANCOVAs controlling for clinic and patient characteristics.

#### 3.4.3. Aim 3 analytic considerations

For survey questions, we will use *t*-tests to compare relative acceptability, appropriateness, and feasibility between the treatment conditions and primary care clinics. Any items that produce averages below the scale midpoint will be addressed through analysis of the focus group and interview data to explore if we can determine aspects of the intervention or the patient or supporter experience that would correspond to those unfavorable ratings. We will also complete mediational analyses as defined in Section 3.4.1 to determine if relational or technique factors as measured by self-report measures identified in Table 2 mediate changes in clinical symptoms or if perceptions of acceptability, appropriateness, or feasibility mediate differences in adoption.

We will use thematic analysis as described by Braun and Clarke. [31] This six-step analytical approach facilitates the process of becoming familiar with the data, systematically identifying individual codes, grouping those codes into preliminary themes, defining and naming the final themes that commonly occurred across the entire data set, and then selecting examples from the data to accurately illustrate each theme. We will use consensus to ensure the analytic narrative represents the data, in relation to the research questions. In our consensus process, all reviewers will independently code all of the transcripts and meet to compare their coding to arrive at consensus judgments through open dialogue [54,55].

## 4. Discussion

This project aims to evaluate the effectiveness and implementation of Spanish language dCBT for LEP Latinx patients with depression or anxiety recruited from safety-net primary care clinics. We will compare the effects of a peer-supported version of the dCBT versus an unsupported version. Further, we will test the impact of two implementation strategies: outreach direct to patients versus inreach through provider referrals. Lastly, we will examine mechanisms of change for the intervention and implementation approach.

This study can fill important gaps in the literature and clinical practice. Although the superiority of supported over unsupported dCBT has been demonstrated such that supported dCBT is generally recommended [51,56], investigating whether this support can be provided by community members has not been adequately studied. Findings from this study could help establish a model of support that could be more scalable, especially for traditionally underserved populations. Specific implementation strategies to integrating dCBT into care settings have not been established [19] and are necessary to understand for dissemination into clinical practice. Clear enthusiasm exists for direct-to-consumer type approaches for digital health to improve reach, but the effectiveness of such approaches is unclear. Few studies have addressed the implementation of dCBT, especially in safety-net settings. Studies that have explored implementation have not compared different implementation strategies or worked specifically with LEP Latinx [57].

### 4.1. Limitations

Despite the strengths of the SUPERA study, several limitations are worth noting. We do not examine all aspects of implementation, but focus on select elements from the RE-AIM model. Although we are considering cost, we are not doing a full cost-effectiveness analysis. Lastly, we will examine sustainment during this trial, which is focused on initial implementation.

### 4.2. Future directions

We anticipate a few future directions that could stem from this work. First, if successful, we could look at the potential for sustainment in the clinics where dCBT is implemented. Subsequent work could also examine the potential to both scale up [58] and scale out this intervention [59]. Scaling up could examine implementation in other primary care settings for LEP Latinx, using the implementation strategy identified as most successful. Scaling out could explore implementation in other settings, for example, community-based settings, or with other populations where culturally and linguistically tailored interventions and support might be useful to deliver dCBT. All of this work could help determine how extensible our findings would be in new settings and populations.

### 4.3. Conclusion

We will never have enough mental health professionals to meet the burden associated with depression and anxiety, especially for historically marginalized groups like Latinx population. Furthermore, traditional evidence-based practices for mental health are resource intensive and often not available for people from disenfranchised groups such as those with LEP. This study tries to overcome these challenges by evaluating the implementation of dCBT in safety-net primary care clinics for a population, LEP Latinx, that have few available options for mental health treatments. The use of community peers as supporters of a dCBT platform could dramatically improve its effectiveness, adoption, and implementation in low-resource settings. These innovations could have broad benefits, creating sustainable, scalable solutions that meet the needs of other settings and populations and ultimately expand to target a wider range of health and mental health problems.

## Figures and Tables

**Table 1:** Outcomes organized by RE-AIM framework.

Aim	Dimension definition	Application to project	Study Measurement(s)
**Aim 2**	**Reach:** Proportion of the target population that participated in the intervention	Percentage of eligible individuals contacted and onboarded to the dCBT platform; representativeness of the overall target population of LEP Latinx patients.	1) Numerators equal to number of patients a) contacted and b) who create account with the denominator equal to the number eligible as identified by the Patient Registry. 2) Characteristics of participants compared to nonparticipants (by age, gender, etc.)
**Aim 1**	**Effectiveness:** Success rate if implemented as planned	Improvements in symptoms and functioning	(PHQ-9, GAD-7, and WHODAS: 8 weeks (treatment end) and a 3-mo follow-up.
**Aim 2 and 3**	**Adoption:** Number of setting and people who are willing to initiate the program	Measuring the integration of dCBT into primary care clinics	Percent of providers with at least one enrolled patient; characteristics of providers with at least one enrolled patient; use of qualitative methods to understand adoption at setting and staff level (Aim 3)
**Aim 2 and 3**	**Implementation:** Extent to which intervention is implemented as intended in the real world	Tracking of the fidelity of the intervention to the protocol and costs associated with implementing as well as implementation outcomes (acceptability, appropriateness, feasibility)	Cost of delivery (Aim 2). Tracking of peer fidelity and interactions. Implementation climate, organizational readiness for change, and intervention acceptability, appropriateness, and feasibility; Qualitative interviews outlining contextual and process factors (Aim 3).

**Table 2:** Measures and outcomes to evaluate mechanisms of implementation strategies.

Aim	Target	Assessment	Measure	Outcome
3a	Patient	Interview	Semi-Structured Interview Guide	Acceptability, feasibility, appropriateness, cultural relevance of dCBT
3a	Patient	Survey	Supportive Accountability Measure [60] 12/22/2023 1:48:00 PM	Relationship factors of bond, accountability, and legitimacy
3a	Patient	Survey	Working Alliance Inventory	Relationship factors of Bond/working alliance
3a	Patient	Survey	Frequency of Actions and Thoughts Scale [61]	Technique factors of CBT skill use
3a	Patient	Survey	Knowledge Gain in dCBT [62]	Technique factors of knowledge of CBT skills
3a	Patient	Survey	Acceptability, Appropriateness, Feasibility Measure [63]	Acceptability, feasibility, and appropriateness of dCBT
3a	Supporters	Observation	Fidelity Monitoring and Checklist	
3b	Providers/Leadership	Interview/Focus Groups	Semi-Structured Interview Guide	Acceptability, feasibility, appropriateness, clinical relevance of dCBT
3b	Providers/Leadership	Survey	Acceptability, Appropriateness, Feasibility Measure [63]	Acceptability, feasibility, and appropriateness of dCBT
3b	Providers/Leadership	Survey	Implementation Climate Scale [64]	Organizational climate that could support implementation
3b	Providers/Leadership	Survey	Organizational Readiness for Change [65]	Strength of evidence for change, quality of organizational context, organizational capacity and resources

## Data Availability

No data was used for the research described in the article.
